# Early intervention increases reactive joint attention in autistic preschoolers with arousal regulation as mediator

**DOI:** 10.1007/s00787-025-02738-1

**Published:** 2025-05-10

**Authors:** Nico Bast, Leonie Polzer, Naisan Raji, Luisa Schnettler, Solvejg Kleber, Christian Lemler, Janina Kitzerow-Cleven, Ziyon Kim, Marie Schaer, Christine M. Freitag

**Affiliations:** 1 Department of Child and Adolescent Psychiatry, Psychosomatics and Psychotherapy, Autism Research and Intervention Center of Excellence, University Hospital Frankfurt, Goethe-University, Deutschordenstraße 50, 60528 Frankfurt am Main, Germany; 2 Department of Psychiatry, University of Geneva, Geneva, Switzerland

**Keywords:** Pupillometry, Eye tracking, Biomarker, Gaze following, Neurodiversity, NDBI

## Abstract

Reactive joint attention (RJA) describes shared attention on a cued target. This key ability is attenuated in autistic compared to non-autistic preschoolers with low cognitive ability, and thus trained during early intervention. We evaluated the development of RJA in matched autistic preschoolers within a randomized controlled trial of the naturalistic developmental behavioral intervention A-FFIP (intervention [*n* = 32] versus early intervention-as-usual [EIAU, *n* = 28]), which is further compared to non-autistic preschoolers (*n* = 52). A screen-based eye-tracking paradigm assessed RJA at baseline, after 12 months (end-of-intervention, 78% retention), and after 36 months (follow-up, 44% retention). Corresponding pupil size changes were utilized to investigate arousal as a mediator in RJA group differences. Generalized linear mixed models were applied to compare RJA likelihood between groups and assessment timepoints. Across timepoints, RJA likelihood was lower in autistic versus non-autistic preschoolers (ORs = 0.07–0.27). The A-FFIP - but not the EIAU group - showed an increase in RJA likelihood at end-of-intervention (OR = 1.52) and follow-up (OR = 2.38). Across both autistic groups, an increase in RJA likelihood after 12 months predicted improved social responsiveness at 36-months follow-up (β = -1.22). A higher baseline pupil size within trials was associated with a lower RJA likelihood (β = -0.32) and mediated the autistic group difference on RJA likelihood in a causal mediation analysis. The A-FFIP early intervention increased eye-tracking derived RJA in autistic preschoolers up to two years after end of intervention, which likely cascaded on improved social responsiveness. Arousal regulation is outlined as a promising mediating mechanism.

**Supplementary Information:**

The online version contains supplementary material available at 10.1007/s00787-025-02738-1.

## Introduction

Joint attention (JA) refers to shared attention between two individuals on a stimulus. In non-autistic infants, it occurs around age 9–12 months [1]. JA integrates orienting attention to social agents, identifying communicative signals, and utilizing re-orienting cues to a target [2]. JA encourages inferences on intentions and facilitates social learning [3], and is a prerequisite for the development of a Theory of Mind (ToM) [4]. JA has been differentiated into reactive joint attention (RJA) as responding to re-orienting cues, and initiating joint attention (IJA) as active initiation by the child [5]. RJA is sometimes used synonymously to gaze following [6] but also gestures such as pointing may serve as cue [7]. Gaze following in infancy predicted utilization of mental-state words (indicating ToM) as toddlers which develops into broader social cognition as preschoolers [8]. This represents a developmental cascade that differs in autism [9].

Autism spectrum disorder (ASD) is a neurodevelopmental condition characterized by limited social communication and restricted and repetitive behaviors. Attenuated joint attention has been established in autistic preschoolers [2]. An early longitudinal study in autistic children associated IJA at 20 months-of-age with better language ability and lower symptom severity at 42 months-of-age [7]. In contrast, attenuated RJA has been reported specifically for low nonverbal IQ in autistic versus non-autistic preschoolers [10, 11]. Others have questioned that attenuated RJA compared to IJA is predictive of a later autism diagnosis [3, 12]. Recently, RJA in autistic preschoolers has been shown to predict the growth rate of expressive language over the course of 9 months [13]. Meta-analyses in autistic and non-autistic children supported cross-sectional associations of RJA and IJA with receptive and expressive language ability [14], and of RJA with questionnaire measures of social functioning [15]. Previously, a prospective effect of RJA on socio-cognitive development has only been shown in non-autistic children [16, 18]. Here, we will assess the prospective effect of RJA on the development of social responsiveness in autistic and non-autistic preschoolers.

RJA is often targeted in autism-specific early interventions for preschoolers [19]. A pioneering study emphasized joint attention (combined RJA and IJA) as a mediating mechanism in the association of social attention and development of language [20]. A meta-analysis on early interventions in autism explicitly training joint attention reported a moderate joint attention increase at end-of-treatment [21]. Naturalistic, Developmental, Behavioral Interventions (NDBI) represent a type of early intervention that utilizes natural contingencies in reinforcement learning to teach developmentally appropriate skills like joint attention in a naturalistic learning environment [22]. A recent systematic review showed that NDBI are the only type of early intervention that led to a reduction in autism core symptoms when controlling for studies’ risk of bias [23]. A prominent NDBI is the JASPER (joint attention, symbolic play, engagement and regulation) intervention, for which RJA (labelled as joint engagement) has been shown to mediate the intervention effect on IJA improvements [24]. This suggests RJA as a fundamental mechanism of change in early interventions. The Frankfurt Early Intervention Program for Autism Spectrum Disorder (A-FFIP) is novel, manualized NDBI of low intensity that can be scaled in local health care systems [25]. The current study investigates the effect of A-FFIP or early intervention as usual (EIAU), provided over one year, on the longitudinal development of RJA in autistic preschoolers.

Joint attention can be assessed by direct observation of the child’s behavior. For example, the Early Social Communication Scales (ESCS) are applied to rate RJA and IJA in videorecorded observations of structured social interactions [26]. Eye-tracking based paradigms provide a more standardized and time-economic assessment of RJA [27]. Still, eye-tracking paradigms on gaze following as RJA measure often failed to elicit differences between autistic and non-autistic children [2], a finding contrasting with the results of a live social interaction paradigm [28]. These null findings in eye tracking were mostly reported when RJA was analyzed by an aggregated difference measure in fixed effects models [29, 31]. Given the high interindividual heterogeneity in autism phenotypes [32], such an aggregated difference measure provides subpar statistical power to capture between-group differences [33]. This power might be improved by the application of mixed models that consider interindividual variability [34].

To date, neurophysiological mechanisms underlying RJA in autistic preschoolers remain elusive. Recently, atypical early-stage processing has been proposed as mediator of genetic risk on the behavioral phenotype in autism [35]. This early-stage processing can be assessed during joint attention paradigms by pupillometry [36]. Pupillometry provides a baseline pupil size (BPS) and a stimulus-evoked pupillary response (SEPR) [37, 38]. BPS is an index of neurophysiological arousal [39] that modulates higher-order cognitive processes including attention [40, 41]. SEPR is inversely related to BPS [42] and represents an index of stimulus-specific processing in response to salience and utility [43, 44]. The Locus Coeruleus – Norepinephrine (LC-NE) system is one primary driver of pupillary responses (BPS and SEPR) [41]. We showed pupillometric markers of LC-NE activity as predictors of attentional performance [45], where increased SEPR towards unexpected stimuli differentiated between autistic and non-autistic children. Other studies reported generally increased BPS [46] or decreased BPS [47] in autistic compared to non-autistic preschoolers. In an experimental manipulation of task utility, we showed an attenuated adaptation of BPS and SEPR in autistic versus non-autistic children, which was interpreted as a different arousal regulation in autism [48]. It supported previous findings that suggested different arousal as a mechanism of altered attention in autistic children [49, 51]. Recent empirical work in nonautistic infants outlined arousal (as measured by heart rate) as a mediator of gaze following [52]. Here, we evaluate pupillometric markers of arousal regulation as underlying mechanism of between-group differences in RJA.

The current study aims to investigate the effect of an NDBI on the development of RJA in autistic preschoolers. RJA is investigated with an eye-tracking paradigm. In intervention models, we compare an intervention (A-FFIP) versus an early intervention-as-usual (EIAU) group concerning RJA at baseline, after 12 months (end-of-intervention), and after 36 months (follow-up). In developmental models, we compare the autistic groups versus non-autistic preschoolers concerning RJA at baseline and follow-up. Generalized linear mixed models are applied to assess RJA likelihood per trial, which controls for interindividual variability. We expect lower RJA likelihood in autistic compared to non-autistic preschoolers (hypothesis 1). We hypothesize that A-FFIP versus EIAU improves RJA likelihood in autistic preschoolers (hypothesis 2). We further propose a positive effect of RJA change at end-of-intervention on social responsiveness at follow-up (hypothesis 3). We also expect that the intervention might change pupillometric measures (hypothesis 4). Lastly, we hypothesize changes in pupillometric measures (BPS, SEPR) to mediate group differences in joint attention (hypothesis 5). This would emphasize arousal regulation as a process-outcome mechanism of RJA development.

## Methods

### Sample

The final sample (*n* = 112) consisted of *n* = 32 autistic preschoolers receiving A-FFIP, *n* = 28 autistic preschoolers receiving EIAU, and *n* = 52 non-autistic preschoolers (see Table 1). Trained staff confirmed an autism spectrum disorder (ASD) according to DSM-5 diagnostic criteria using the German versions of the Autism Diagnostic Observation Schedule 2 (ADOS-2) [53] and the Autism Diagnostic Interview-Revised (ADI-R) [54] or the ADI-R toddler algorithm [55]. Autistic preschoolers were part of the Frankfurt subgroup of the multicenter, parallel-group, randomized controlled trial on the A-FFIP program [25]. In a study addon, non-autistic preschoolers were recruited by local advertisement in kindergartens, social media and health system institutions. Detailed inclusion and exclusion criteria have been described previously [56].

**Table 1:** Sample description: autistic and non-autistic groups at baseline and follow-up

	timepoints	A-FFIP - autistic individuals	EIAU - autistic individuals	non-autistic individuals
timepoints (baseline/follow-up)		32 / 11	28 / 12	52 / 26
biological sex (female/male)		6 / 26	4 / 24	16 / 36
age (m)	baseline	49.6/11.1 [26.63–65.57]	47.58/9.17 [31.4-66.47]	35.85/14.07 [18.33–73.43]
	follow-up	86.12/13.22 [64.67–104.5]	84.11/7.53 [73.23–98.87]	70.97/22.95 [35.37-118.23]
developmental age (m)	baseline	28.77/10.62 [16.11–65.08]	28.27/8.97 [16.52–52.95]	35.99/14.68 [16.05–76.61]
	follow-up	48.74/35.27 [23.84–104.5]	56.13/31.59 [24.76–95.26]	57.49/16.02 [30.55–85.92]
nonverbal IQ	baseline	59.53/19.22 [31–101]	60.94/17.12 [37–108]	102.1/12.79 [73–128]
	follow-up	53.22/36.91 [26–112]	65.75/34.85 [30–108]	103.07/11.55 [79–121]
SRS-16 total	baseline	27.63/8.15 [8–40]	28.1/6.25 [13–39]	4.6/3.13 [0–12]
	follow-up	27.21/9.32 [10–43]	31.55/7.15 [22–44]	4.12/3.46 [0–12]
RBSR total	baseline	32.12/22.21 [2–93]	42.39/25.92 [10–126]	8.88/9.26 [0–37]
	follow-up	33.36/13.35 [14–54]	53/24.71 [27–102]	6.12/9.51 [0–42]
CBCL total (t-score)	baseline	64.68/9.78 [46–93]	66.46/9.36 [52–96]	48.08/8.54 [27–64]
	follow-up	63.85/6.61 [54–76]	65.88/13.51 [47–87]	48.75/7.99 [36–68]
ADOS CSS	baseline	6.84/1.61 [4–10]	7.21/1.47 [4–10]	-
	follow-up	6.82/1.72 [5–10]	7.5/1.57 [5–10]	-
RJA likelihood (%)	baseline	21.68/21.88 [0-81.25]	13.17/16.95 [0-68.75]	42.19/31.99 [0-100]
	follow-up	34.66/26.86 [0–75]	17.19/20.32 [0-62.5]	52.84/25.05 [0-93.75]
RJA duration (s)	baseline	0.34/0.19 [0.05–0.7]	0.34/0.19 [0.1–0.93]	0.43/0.19 [0-0.92]
	follow-up	0.41/0.16 [0.24–0.65]	0.4/0.18 [0.17–0.7]	0.43/0.14 [0.15–0.65]
baseline pupil size (mm)	baseline	4.23/0.51 [2.58–5.27]	4.42/0.49 [3.42–5.68]	4.24/0.48 [3.45–5.85]
	follow-up	4.05/0.71 [2.36–5.26]	4.41/0.36 [3.86–4.88]	4.21/0.54 [3.53–5.94]
stimulus-evoked pupillary response (mm)	baseline	0.06/0.17 [-0.37-0.43]	0.06/0.27 [-0.64-0.6]	0.15/0.22 [-0.4-0.78]
	follow-up	0.07/0.24 [-0.43-0.36]	0.24/0.31 [-0.14-1.07]	0.14/0.16 [-0.26-0.44]
total fixation duration (s)	baseline	8.19/1.11 [4.64–9.86]	7.96/1.2 [5.42–10.23]	8.97/1.06 [6.41–10.74]
	follow-up	8.27/1.64 [3.84–9.6]	8.1/1.15 [5.63–9.29]	9.26/0.87 [6.56–10.56]
mean missing data (%)	baseline	23.07/10.36 [2.03–38.79]	26.64/10.98 [6.64–47.57]	21.89/12.98 [1.02–48.45]
	follow-up	25.86/8.95 [13.87–42.67]	28.09/7.87 [16.55–43.41]	16.7/7.99 [3.2-29.65]

Note: Mean / SD [Min - Max]

A-FFIP and EIAU groups were matched at baseline regarding age, biological sex distribution, nonverbal IQ, autism symptom severity (ADOS-2 calibrated severity score), comorbid psychopathology (CBCL 1.5-5) [57], and eye-tracking data quality. The additional group of non-autistic preschoolers were on average 12 months younger than the autistic children to achieve a comparable developmental age across autistic and non-autistic groups. This resulted in a non-autistic group with a younger age, higher nonverbal IQ, and coincidentally a higher proportion of biological girls compared to the autistic groups (A-FFIP and EIAU).

### Procedure

Caretakers provided written informed consent for study participation (ethics approval reference: autistic preschoolers [10/18], non-autistic preschoolers [361/18]). Eye tracking was done in a child-friendly lab with constant artificial lighting. The eye tracker was a Tobii TX300 at 300 Hz sampling rate. Participants sat on a highchair or the caregiver’s lap in front of the presentation screen (27 inches, 1920*1080 pixels) with no restrictions on head movement. Participants were instructed to focus on the screen. A child-friendly five-point calibration was performed. The reactive joint attention (RJA) paradigm took 3 min to complete and was part of a larger eye-tracking battery (25 min, see github). Findings from different paradigms of this battery have been published with partially overlapping samples [h^56^, ^58^].

With eye tracking, autistic preschoolers were assessed three times and non-autistic preschoolers were assessed two times. Autistic preschoolers (A-FFIP and EIAU group) were assessed at baseline, after 12 months (SD = 1.5 months, end-of-intervention), and after 36 months (SD = 2.0 months, follow-up, Table 2). Non-autistic preschoolers were assessed at baseline and after 36 months (SD = 12.1 months, follow-up). See supplements for dates per individual.

**Table 2:** Sample description: autistic groups at baseline (BL), end-of-treatment (+ 12 M), and follow-up (+ 36 M)

	timepoints	A-FFIP - autistic individuals	EIAU - autistic individuals
timepoints (BL/+12 M/+36 M)		32 / 27 / 11	28 / 20 / 12
biological sex (female/male)		6 / 26	4 / 24
age (m)	baseline	49.6/11.1 [26.63–65.57]	47.58/9.17 [31.4-66.47]
	+ 12 months	64.98/11.39 [47.6-80.43]	60.6/10.1 [44.9-79.03]
	+ 36 months	86.12/13.22 [64.67–104.5]	84.11/7.53 [73.23–98.87]
nonverbal IQ	baseline	59.53/19.22 [31–101]	60.94/17.12 [37–108]
	+ 12 months	56.24/23.6 [28–110]	59.24/20.84 [34–114]
	+ 36 months	53.22/36.91 [26–112]	65.75/34.85 [30–108]
SRS-16 total	baseline	27.06/8.3 [8–40]	28.57/6.17 [15–39]
	+ 12 months	24.19/8.84 [4–39]	27.54/6.27 [13–36]
	+ 36 months	26.9/10.35 [10–43]	33.11/6.86 [25–44]
RBSR total	baseline	28/17.77 [2–67]	47.05/27.95 [10–126]
	+ 12 months	29.38/23.13 [3–92]	42.54/27.84 [8–87]
	+ 36 months	35.9/13.5 [14–54]	57.11/25.66 [27–102]
CBCL total t-score	baseline	63.47/7.99 [46–82]	66.96/10.19 [52–96]
	+ 12 months	63.38/14.38 [40–93]	65.77/8.8 [53–82]
	+ 36 months	66/5.87 [58–76]	67.5/14.94 [47–87]
ESCS RJA (%)	baseline	51.1/28.09 [7.14–100]	44.37/27.83 [0-100]
	+ 12 months	-	-
	+ 36 months	-	-
RJA likelihood (%)	baseline	21.68/21.88 [0-81.25]	13.17/16.95 [0-68.75]
	+ 12 months	26.62/18.8 [0-68.75]	19.38/22.75 [0-81.25]
	+ 36 months	34.66/26.86 [0–75]	17.19/20.32 [0-62.5]
RJA duration (s)	baseline	0.34/0.19 [0.05–0.7]	0.34/0.19 [0.1–0.93]
	+ 12 months	0.3/0.11 [0.07–0.46]	0.28/0.14 [0.15–0.61]
	+ 36 months	0.41/0.16 [0.24–0.65]	0.4/0.18 [0.17–0.7]
baseline pupil size (mm)	baseline	4.23/0.51 [2.58–5.27]	4.42/0.49 [3.42–5.68]
	+ 12 months	4.29/0.55 [2.51–5.19]	4.28/0.49 [3.26–5.2]
	+ 36 months	4.05/0.71 [2.36–5.26]	4.41/0.36 [3.86–4.88]
stimulus-evoked pupillary response (mm)	baseline	0.06/0.17 [-0.37-0.43]	0.06/0.27 [-0.64-0.6]
	+ 12 months	0.06/0.2 [-0.28-0.46]	0.06/0.2 [-0.23-0.53]
	+ 36 months	0.07/0.24 [-0.43-0.36]	0.24/0.31 [-0.14-1.07]
total fixation duration (s)	baseline	8.19/1.11 [4.64–9.86]	7.96/1.2 [5.42–10.23]
	+ 12 months	7.97/0.97 [5.72–10.03]	7.56/1.82 [3.79–10.31]
	+ 36 months	8.27/1.64 [3.84–9.6]	8.1/1.15 [5.63–9.29]
mean missing data (0–1)	baseline	23.07/10.36 [2.03–38.79]	26.64/10.98 [6.64–47.57]
	+ 12 months	26.27/8.52 [10.9-41.48]	26.86/12.14 [1.85–47.3]
	+ 36 months	25.86/8.95 [13.87–42.67]	28.09/7.87 [16.55–43.41]

Note: Mean / SD [Min - Max]

**Fig. 1:**
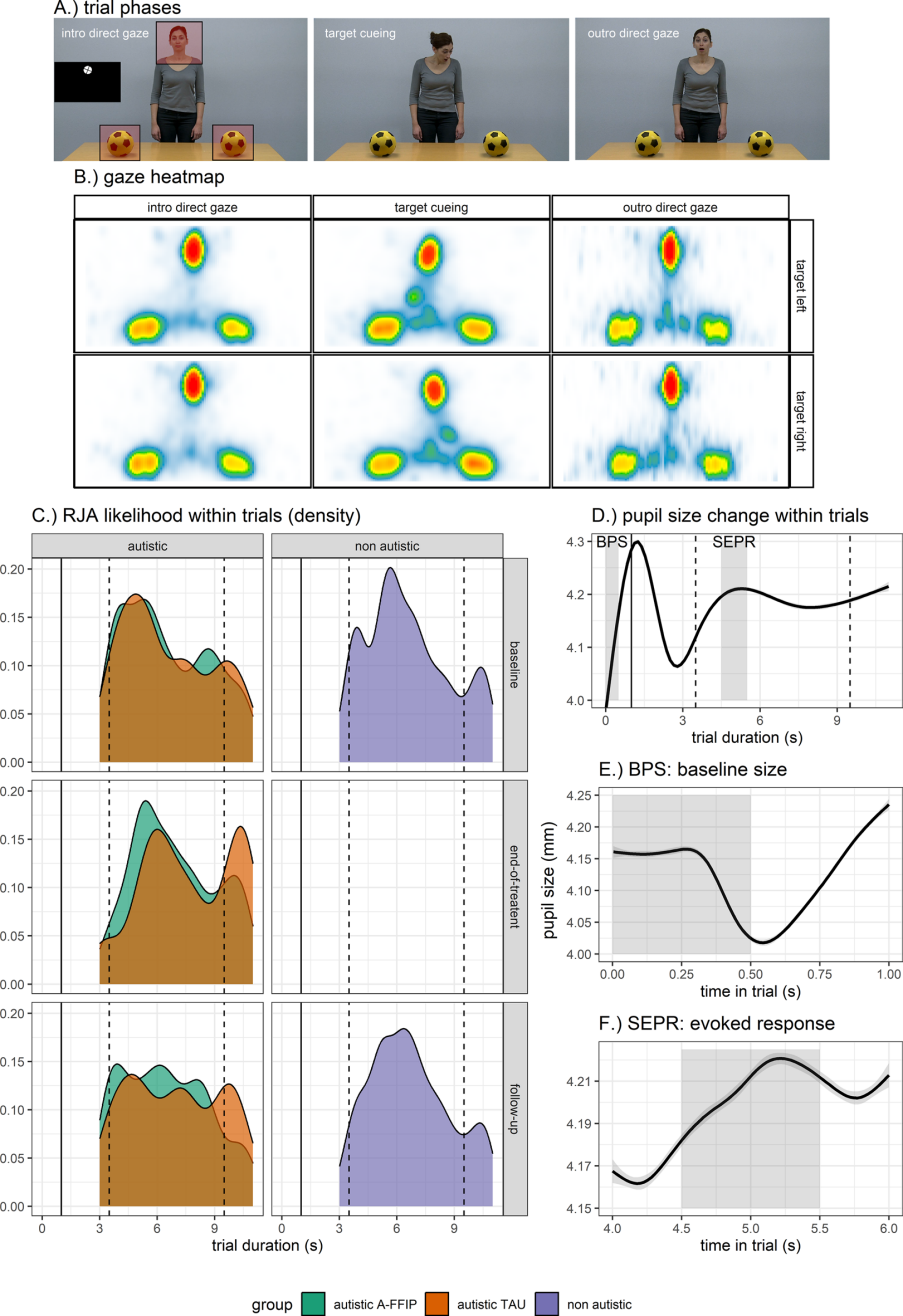
Stimulus Material and main measures. (**A**) Phases of trials. The initial attention grabber phase (1s) is displayed as insertion in the left picture. The left picture also shows the area-of-interest (AOIs) definition across phases. (**B**) Heatmap of gaze fixation data between trial phases. Online Supplements further provide a heatmap animation of gaze fixation data within trials to illustrate dynamic gaze behavior. (**C**) Reactive joint attention (RJA) likelihood within trials displayed as density (occurrences / observations) between groups and timepoints. The solid vertical line indicates the end of the attention grabber phase and start of the video. Dashed lines show the onset and offset of the target cueing phase. (**D**) Pupil size change within trials. An initial increase and decrease is associated with luminance adaptation to the attention grabber stimulus that was displayed on a dark background. (**E**) Baseline pupil size (BPS) – Zoom-in on time span used for calculation (0–0.5 s) and shaded in grey. It shows that the BPS is not influenced by the luminance adaptation. (**F**) Stimulus-evoked pupillary response (SEPR) – Zoom-in on time span used for calculation (4.5–5.5) and shaded in grey. Dilation corresponds to the onset of target cueing

### Additional measures

In autistic and non-autistic preschoolers, we assessed nonverbal IQ (Bayley-III, WPPSI-III) [59, 60], social responsiveness (SRS-16) [61], and comorbid psychopathology (CBCL-1.5-5).

Autistic preschoolers participated in additional assessments according to the A-FFIP study protocol [25]. This included the Early Social Communication Scales (ESCS) [26] at baseline, from which the RJA subscale was utilized to validate the RJA eye-tracking task. The ESCS (high-level) RJA subscale represents the likelihood (0-100%) of the child to respond to cued targets in a live gaze following task with pointing. The ESCS RJA subscale was coded based on video recordings by blinded raters (ICC = 96.6 [95%-CI 96.4 to 96.7]). Also only in autistic preschoolers, we assessed a verbal IQ (Bayley-III, WPPSI-III), which provided a right skewed distribution (median = 22.5, mean = 33.3) due to floor effects. Thus, verbal IQ was not considered in the statistical analysis.

### Stimuli

The RJA eye-tracking paradigm is a gaze following task that varies the intensity of facial expressions (neutral, mild, intense) between cueing conditions and includes pointing as an additional cueing condition [62]. The paradigm consists of 16 trials that are presented in a pseudorandom order, in two blocks. Each trial has a duration of 11s with different phases (see Fig. 1A): An initial attention grabber at the location where a human face will appear (1s; a rotating black fixation cross on a white circle on a black background, see Fig. 1A left), intro direct gaze: a human looks directly into the camera and two stimuli are presented (2s), target cueing: the human initiates target cueing to one target stimulus as a gaze on the target paired with a facial expression or pointing (6s), outro direct gaze: the human looks into the camera again maintaining the facial expression (2s). Trials differ by target cueing (neutral gaze, mild facial expression, intense facial expression, neutral gaze + pointing) and stimulus (rabbit, truck, ball, flower). The position of the target stimulus (left, right) was counterbalanced between trials.

### Data preprocessing

Raw eye-tracking data were preprocessed with R statistics (4.3, see github) according to peer-reviewed guidelines for gaze [63] and pupillometry data [38]. Data were segmented per trial. A blink correction excluded eye blinks between 75 and 250 ms and 25 ms before and after. Trials with less than 50% of data were dropped.

#### Reactive joint attention (RJA)

Gaze data points beyond plausible ranges were dropped (velocity > 1000 degrees of visual angle per second [°/s], acceleration > 10000 °/s). Gaze data were smoothed with a Savitzky-Golay filter with a length of 70 ms. Saccades were identified by a velocity-based algorithm with data-driven thresholds that consider intra- and interindividual differences in data noise [63]. Fixations were identified by the absence of saccades and a gaze position change smaller than 1 °/s for at least 100 ms (Fig. 1B).

RJA was defined similarly to the original study that designed the paradigm [62] as when (1) a fixation on the head of the presented human occurred within the last 500 ms and (2) was followed by a fixation on the target object in the target cueing phase or after. The area-of-interest definition is shown in Fig. 1A. We deviated from the original definition as we did not consider a distinct hand area-of-interest for the pointing condition to retain comparable RJA definitions across target cueing conditions. For each trial, RJA likelihood was defined as the presence or absence of any RJA in a trial (true versus false; Fig. 1C shows the distribution of RJA likelihood within trials). This eye-tracking-based RJA likelihood was the main outcome measure of the current analysis. RJA likelihood in eye tracking was positively associated with an ESCS-derived RJA in behavioral observations (β = 0.29 [0.04, 0.53], t(59) = 2.29, *p* =.025).

#### Pupillometry (BPS, SEPR)

Data points outside plausible ranges were dropped (< 2| > 8 mm). Pupillometry data were smoothed by a linear filter (< 3 times median absolute deviation) with missing interpolation (150 ms window) [48]. The estimated pupil size was based on the mean of both eyes (*r* =.97, Fig. 1D). A baseline pupil size (BPS) was calculated as a mean pupil size during the first 500 ms of each trial, i.e. the first half of the attention grabber phase (Fig. 1E). Pupil size estimates within trials were normalized by subtracting the respective BPS [37]. For each trial, we estimated a stimulus-evoked pupillary response (SEPR) as mean normalized pupil size between 4500 and 5500ms after stimulus onset, which occurred after the onset of the target cueing (Fig. 1F).

### Statistical analysis

Analyses were done with R statistics (4.3, see github). Preprocessing provided (eye-tracking based) RJA likelihood, BPS, and SEPR measures that were investigated in distinct models on a per-trial level. RJA likelihood (True versus False) was investigated in generalized linear mixed models with a binomial link function to estimate group differences (hypothesis 1, 2). Group (autistic A-FFIP, autistic EIAU, non-autistic) and measurement timepoint (baseline, end-of-treatment [+ 12 months], follow-up [+ 36 months]) and their interaction were included as fixed effects. In intervention models, autistic groups (A-FFIP versus EIAU) were compared at baseline and end-of-treatment. In development models, autistic and non-autistic groups (A-FFIP versus EIAU versus non-autistic) were compared at baseline and follow-up. We differentiated between intervention and developmental models as non-autistic preschoolers had no end-of-treatment assessment (as they received no treatment), which would have led to missing factor level combinations in models including all timepoints. In our models, participant and trial number were included as random intercepts to control for interindividual heterogeneity (see supplements). There were no effects of task conditions including target cueing (neutral, mild, intense, neutral + pointing), stimulus (rabbit, truck, ball, flower), or target position (left, right) on RJA likelihood (all *F*s < 1). Thus, these variables were not considered.

Group differences in RJA likelihood between autistic groups (hypothesis 2) were further supported by sensitivity and dropout analysis to rule out alternative explanations to an intervention effect. Sensitivity analysis as generalized mixed model with the same random effect structure were applied to test whether baseline RJA likelihood influenced RJA likelihood at end-of-intervention and follow-up. Dropout analysis as two-way ANOVA models were applied with group (A-FFIP versus EIAU) and retention status (at end-of-treatment: True versus False; or at follow-up: True versus False) as two-level factors. Dependent variables were RJA likelihood, autism symptom severity (ADOS-2 CSS), comorbid psychopathology (CBCL-1.5-5), or eye-tracking data quality (missing data; see Table 1). These dropout models achieved a power = 0.82 to detect a moderate effect (η² = 0.12) with the given sample.

In linear models, cascade effects were investigated as whether manifest changes in key variables (RJA likelihood, BPS, SEPR) from baseline to end-of-treatment influenced manifest changes in socio-cognitive development from baseline to follow-up (hypothesis 3). Socio-cognitive development was investigated by changes in parent reports of social responsiveness (SRS-16 sum score) [61], restricted and repetitive behavior (RBSR sum score) [64], and nonverbal IQ (Bayley-III or WPPSI-IV).

In linear mixed models, the same fixed and random effect structure as in RJA likelihood models was applied to investigate intervention and developmental effects on BPS and SEPR between groups (hypothesis 4). In causal mediation analysis [65, 66], we tested whether observed group differences in RJA likelihood between A-FFIP and EIAU at end-of-treatment or follow-up are mediated by pupillometry (BPS, SEPR; hypothesis 5). This was controlled for the moderated mediation effect of respective baseline values. It utilizes simulations (k = 1000) to estimate an average causal mediation effect (ACME) that represents the expected difference in RJA likelihood between autistic groups, when pupillometry values across groups would change from EIAU to A-FFIP means. In addition, the average direct effect (ADE) represents the expected difference when the pupillometry measure as mediator is held constant. ADE and ACME add up to the total effect of group on RJA likelihood. Quasi-Bayesian Monte Carlo simulations are applied to estimate 95% confidence intervals.

Mixed model fits were estimated with the marginalized (*mR*²) and conditional (*cR²*) coefficient of determination [67]. In generalized linear mixed models, planned contrasts of RJA likelihood between groups and timepoints were investigated with marginalized means and 95% bootstrapped confidence intervals ([2.5%, 97.5%]) of Odds Ratio (OR). In linear mixed models, planned contrasts of BPS or SEPR between groups and timepoints were investigated with marginalized means and 95% bootstrapped confidence intervals ([2.5%, 97.5%]) of effect size (Δβ).

## Results

### RJA likelihood between groups and timepoints (hypothesis 1,2)

**Fig. 2:**
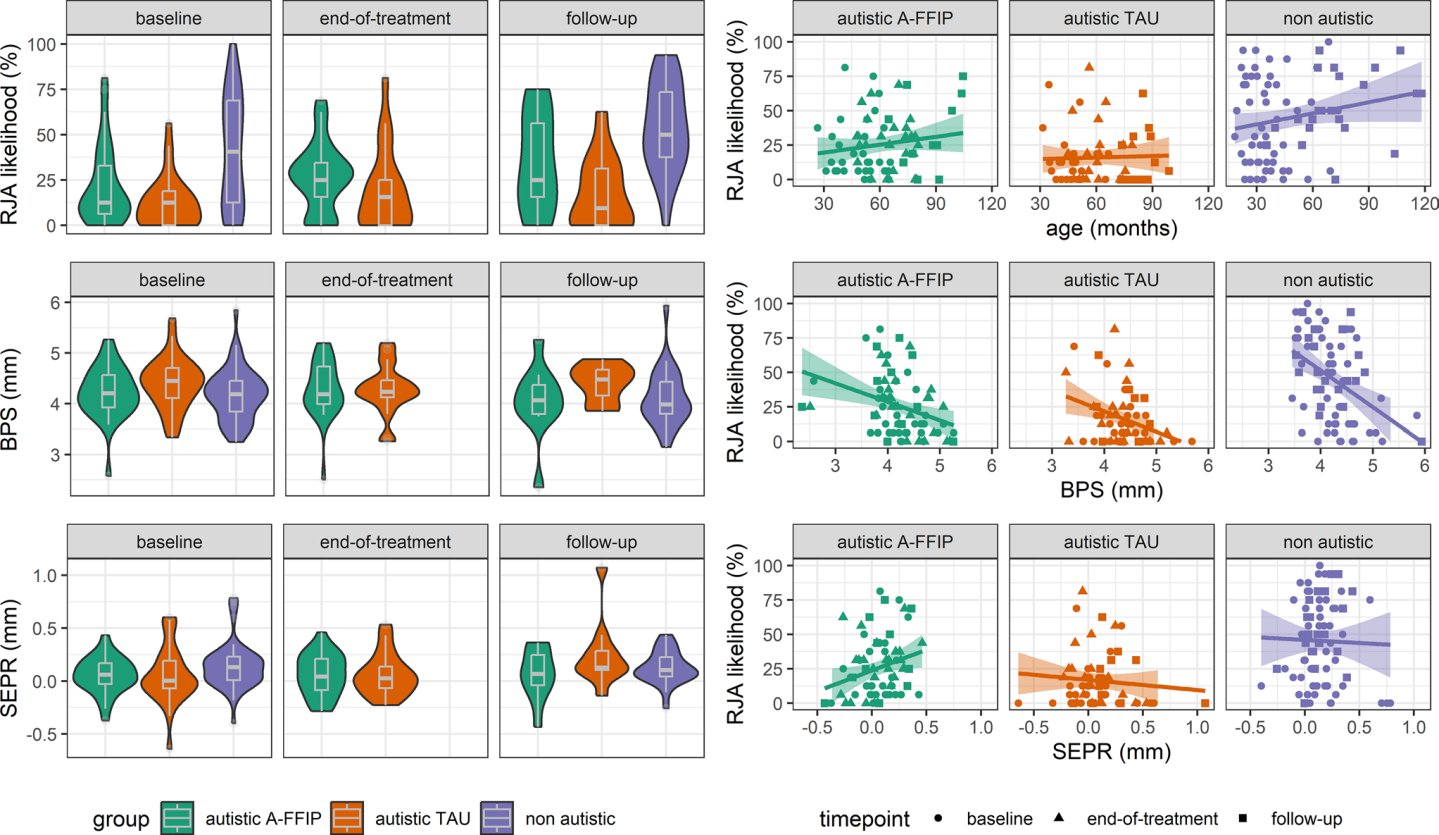
Left: RJA likelihood, baseline pupil size (BPS), and stimulus-evoked pupillary response (SEPR) between groups and measurement timepoints. Violin plots show the density of the measure, while boxplots indicate central tendency as mean and dispersion as interquartile range. Right: Association of RJA likelihood with age, BPS, and SEPR

At baseline, RJA likelihood as an eye-tracking measure descriptively differed between groups (marginalized means; autistic A-FFIP: m = 0.14 [0.08, 0.23], autistic EAIU: m = 0.06 [0.03, 0.11], non-autistic: m = 0.38 [0.26, 0.51]). In the intervention model (*mR*² = 0.05, *cR²* = 0.37), RJA likelihood significantly increased from baseline (BL) to end-of-treatment (ET) in A-FFIP (OR = 1.52 [1.07, 2.18], z = 2.31, *p* =.021), but not in EIAU (OR = 1.49 [0.96, 2.31], z = 1.78, *p* =.075), albeit effect sizes were comparable. At baseline and end-of-treatment, RJA likelihood was higher in A-FFIP compared to EIAU (OR|BL = 2.47 [1.18, 5.18], z = -2.40, *p* =.032; OR|ET: 2.53 [1.18, 5.42], z = -2.38, *p* =.034, Fig. 2).

In the development model (*mR*² = 0.17, *cR²* = 0.51), RJA likelihood increased significantly from baseline (BL) to the 36-months follow-up (FU) in non-autistics (OR = 2.37 [1.72, 3.26], z = 5.26, *p* <.001) and A-FFIP (OR = 2.38 [1.41, 4.04], z = 3.21, *p* =.001), whereas RJA likelihood did not increase in EIAU (OR = 1.64 [0.89, 3.02], z = 1.57, *p* =.115). At follow-up, RJA likelihood was higher in A-FFIP compared to EIAU (OR|FU = 3.68 [1.38, 9.86], z = 2.59, *p* =.009). At baseline and follow-up, RJA likelihood was lower in A-FFIP (OR|BL = 0.27 [0.13, 0.52], z = -3.86, *p* <.001; OR|FU: 0.26 [0.12, 0.59], z = -3.21, *p* =.001) and EIAU (OR|BL = 0.10 [0.05, 0.22], z = -6.05, *p* <.001; OR|FU: 0.07 [0.03, 0.17], z = -5.91, *p* <.001) compared to non-autistics.

### Supporting sensitivity analysis (on hypothesis 1, 2)

Across groups, RJA likelihood at baseline as a predictor did not influence RJA likelihood at end-of-treatment (odds = 1.38 [0.81, 2.35]) or follow-up (odds = 1.28 [0.73, 2.23]). In the intervention model (*mR*² = 0.05, *cR²* = 0.55), this effect did not differ between A-FFIP and EIAU (OR|A-FFIP = 1.29 [0.68, 2.44]; OR|EIAU = 1.49 [0.65, 3.42]). In the development model (*mR*² = 0.09, *cR²* = 0.88), RJA likelihood at baseline predicted RJA likelihood at follow-up only in non-autistics (OR = 1.84 [1.04, 3.25]) and not in A-FFIP (OR = 0.50 [0.19, 1.32]) or EIAU (OR = 2.26 [0.65, 7.84]). Thus, RJA likelihood difference between groups at baseline likely did not explain group differences in RJA likelihood changes at end-of-treatment or follow-up.

### Supporting dropout analysis (on hypothesis 1, 2)

The retention rate for eye-tracking assessments in autistic preschoolers from baseline to end-of-treatment was 84% in A-FFIP and 71% in EIAU, while the retention rate from baseline to follow-up was 34% in A-FFIP, 43% in EIAU, and 50% in non-autistics. In dropout analyses for the autistic groups, no significant fixed effect of or interactions with retention status were found at baseline for the dependent variables of RJA likelihood, autism symptom severity (ADOS-2 CSS), comorbid psychopathology (CBCL-1.5-5), or eye-tracking data quality (see supplements). Thus, autistic preschoolers with longitudinal eye-tracking assessments compared to the children without did not differ on core measures at baseline.

### Cascade effects of RJA changes on later social responsiveness (hypothesis 3)

**Fig. 3:**
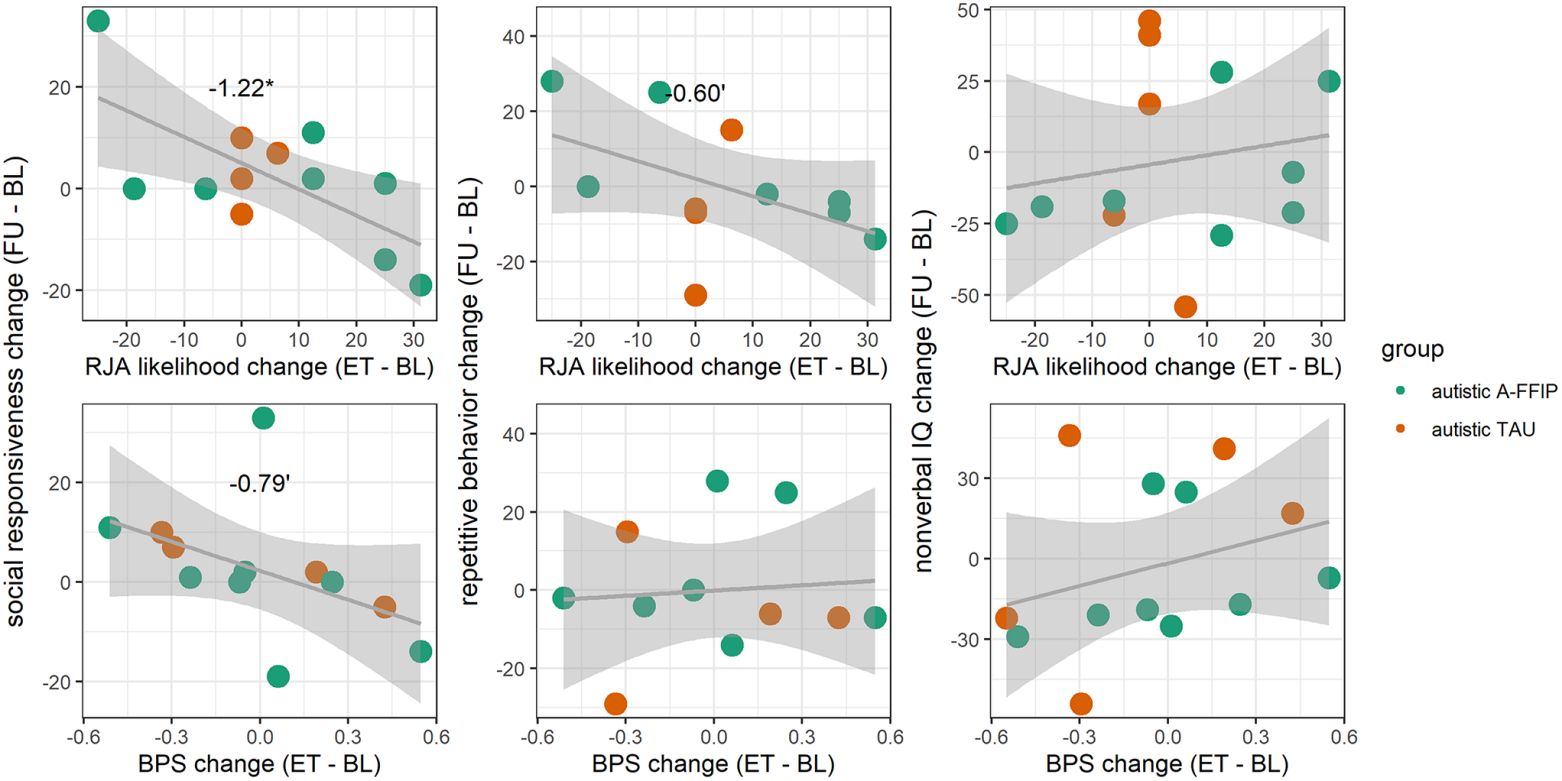
Cascade effects of changes in reactive joint attention (RJA) likelihood or baseline pupil size (BPS) from baseline (BL) to end-of-treatment (ET) on changes in socio-cognitive development from BL to follow-up (FU). Social responsiveness is measured by SRS-16 sum score. Repetitive behavior is measured by RBSR sum score. Nonverbal IQ is measured by Bayley III development quotient or WPPSI-IV nonverbal IQ. *= *p* <.05; ‘ = *p* <.1

In autistic participants with available data at all timepoints, cascading effects were investigated in linear models. Increases in RJA likelihood from baseline to end-of-treatment were associated with an improvement of social responsiveness (i.e. lower SRS-16 total scores) from baseline to follow-up (β = -1.22 [-2.16, -0.29], t(12) = -2.91, *p* =.015). Accordingly, higher RJA likelihood at end-of-treatment predicted higher social responsiveness (i.e. lower scores) at follow-up (β = -0.66 [-1.22, -0.11], t(11) = -2.26, *p* =.024) when controlling for RJA likelihood at baseline. Effects on repetitive behavior and nonverbal IQ were not significant (see Fig. 3).

### Baseline pupil size (BPS) between groups and timepoints (hypothesis 4)

Lower BPS (pupil size during the first 500ms of a trial, Fig. 1E) predicted higher RJA likelihood (β = -0.32 [-0.46, -0.19], z = 4.81, *p* <.001). In an intervention model (*mR*² = 0.01, *cR²* = 0.63), BPS decreased from baseline to end-of-treatment in EIAU (Δβ = -0.17 [-0.29, -0.05]) but not in A-FFIP (Δβ = -0.01 [-0.12, 0.10]). Still, at end-of-treatment, BPS did not differ between the autistic groups (Δβ = 0.05 [-0.36, 0.47], *t* < 1). In a development model (*mR*² = 0.03, *cR²* = 0.64), BPS decreased from baseline to follow-up in the non-autistic group (Δβ = -0.16 [-0.25, -0.07], t(1826) = -3.39, *p* <.001) and A-FFIP (Δβ = -0.28 [-0.43, -0.12], t(1865) = -3.47, *p* <.001), but did not change in EIAU (Δβ = 0.14 [-0.05, 0.33], t(1887) = 2.14, *p* =.146). At baseline, BPS did not differ between groups (*t*s < 1.86, *p*s > 0.154). At follow-up, BPS was lower in non-autistics (Δβ = -0.64 [-1.11, -0.17], t(163) = -3.23, *p* =.004) and A-FFIP (Δβ = -0.62 [-1.14, -0.10], t(175) = -2.80, *p* =.015) compared to EIAU (see Fig. 2).

### Stimulus-evoked pupillary response (SEPR) between groups and timepoints (hypothesis 4)

Overall, higher SEPR was associated with lower BPS (β = -0.88 [-0.92, -0.84], t(1864) = -44.12, *p* <.001). Accordingly, higher SEPR (pupil size change coinciding with target cueing) predicted a higher RJA likelihood across groups (β = 0.13 [0.00, 0.26], z = 2.01, *p* =.044). In an intervention model (*mR*² < 0.01, *cR²* = 0.12), SEPR did not change significantly from baseline to end-of-treatment in the autistic groups (A-FFIP: Δβ = 0.09 [-0.08, 0.25], t(968) = 1.02, *p* =.306; EIAU: Δβ = 0.00 [-0.19, 0.18], t(1005) = -0.02, *p* =.982). In a development model (*mR*² = 0.01, *cR²* = 0.13), SEPR decreased from baseline to follow-up in EIAU (Δβ = -0.18 [-0.29, -0.07], t(975) = -3.13, *p* =.002), and did not change in A-FFIP (Δβ = -0.02 [-0.11, 0.07], t < 1) or non-autistics (Δβ = 0.02 [-0.04, 0.07], t < 1).

### Mediation model of changes in BPS and SEPR on RJA group differences (hypothesis 5)

In causal mediation analysis of the development model with BPS as mediator, a significant total effect of d = 0.23 [0.03, 0.42] (*p* =.028) was observed as the standardized difference in RJA likelihood between A-FFIP and EIAU at follow-up. This total effect consisted of a significant mediating effect of BPS (ACME = 0.09 [0.03, 0.15], *p* <.001) that rendered the direct effect of group to be insignificant (ADE = 0.14 [-0.05, 0.34], *p* =.154). The proportion of mediation by BPS of the total effect was estimated as 0.36 [0.09, 1.52] (*p* =.028). This mediation effect is controlled for a potential moderated mediation effect of BPS at baseline. Thus, the mediation effect of BPS refers to changes of BPS from baseline to follow-up.

A causal mediation analysis for SEPR in the development model did not converge. Causal mediation analysis with BPS or SEPR in the intervention model did not show a significant mediating effect (BPS: ACME = 0.00 [-0.03, 0.04], *p* =.874; SEPR: ACME = 0.00 [-0.01, 0.01], *p* =.500).

## Discussion

This is the first longitudinal study to investigate the development of reactive joint attention (RJA) in samples of autistic and non-autistic preschoolers over the course of three years. This allowed to characterize effects of an early intervention (NDBI as A-FFIP) on changes in RJA likelihood at end-of-intervention or follow-up, and cascading effects on socio-cognitive development at follow-up. We utilized an eye-tracking paradigm to capture RJA likelihood on a per-trial level, which controlled for interindividual heterogeneity and was thus expected to increase the sensitivity to detect group differences. In addition, we utilized pupillometry in eye-tracking to investigate arousal regulation as a mediating mechanism of RJA likelihood.

In a comparison of autistic and non-autistic preschooler, we found a lower RJA likelihood across timepoints (hypothesis 1), which extends a previous cross-sectional finding [62]. These group differences contrast a literature review that emphasized previous null findings in eye-tracking paradigms on gaze following [2]. We showed that this discrepancy is unlikely to be driven by the inclusion of a pointing condition [68]. We rather propose that the consideration of interindividual heterogeneity in RJA likelihood by random intercepts in per-trial analyses (see supplements) increased the sensitivity to detect group differences. The lower RJA in autistic versus non-autistic children in an eye-tracking paradigm extends previous studies of live social interactions in infancy, in which delayed RJA abilities were indicative of a later ASD diagnosis [28] and lower RJA differentiated between children with a high versus low familial association with autism [69]. It emphasizes the application of the RJA eye-tracking paradigm [62] as a time-economic diagnostic measure of prognostic relevance in the preschool age. However, in contrast to the original investigation of this eye-tracking task [62], we did not replicate a modulation of RJA likelihood in autistic preschoolers by the target cueing condition. This might be attributable to a harmonized definition of areas-of-interest across target cueing condition in our analysis or our different analytical approach with a per-trial analysis.

As a main finding, the early intervention (A-FFIP) compared to EIAU was associated with increases in RJA likelihood from baseline to end-of-treatment and a higher RJA likelihood at follow-up (hypothesis 2). Supporting analyses showed that these changes in RJA likelihood were independent of RJA likelihood at baseline and without systematic dropouts. Across groups, the increase in RJA likelihood at end-of-intervention was associated with increased social responsiveness at follow-up (hypothesis 3). Together, we conclude that A-FFIP initiates a developmental cascade in which individual increases in RJA likelihood stabilize beyond the intervention and improve social responsiveness two years after the end of intervention. This has three main implications for the field: First, it emphasizes the prognostic relevance of RJA in autistic development beyond infancy [28]. Second, it provides longitudinal evidence that improved RJA not only influences language development [14] but also socio-cognitive development [15]. Third, it indicates that the positive effect of A-FFIP as an NDBI on social communication and diagnostic characteristics of autism [23] is mediated by improved RJA [21]. This extends recent findings that showed RJA as mediator in the effect of another NDBI (JASPER) on improved initiating joint attention [24], which emphasizes RJA as a change process explaining early intervention effects in autism.

Pupillometry was utilized to investigate arousal regulation as an underlying mechanism of RJA likelihood (hypothesis 4,5). This was based on a recent theoretical model that proposed pupil dilation as an index of different early-stage processing contributing to autistic phenotypes [35]. Baseline pupil size (BPS) is indicative of neurophysiological arousal and stimulus-evoked pupillary response (SEPR) – here in response to target cueing – is indicative of stimulus-specific processing. BPS and SEPR have been associated with the activity of the LC-NE system [70] that modulates attentional processes [71, 72] that are relevant to joint attention. Recent pupillometry findings further suggested different LC-NE functioning in autism with an attenuated SEPR to deviant stimuli during sensory processing [47] and attenuated downregulation of BPS and upregulation of SEPR in response to increased task utility [48].

We replicated a strong inverse association of BPS and SEPR [73]. Importantly, a lower BPS and higher SEPR were associated with a higher RJA likelihood across groups. Between groups, BPS decreased from baseline to follow-up in the autistic A-FFIP and the non-autistic group compared to the autistic EIAU group, and resulted in a lower BPS at follow-up in the autistic A-FFIP and non-autistic group compared to the autistic EIAU group (hypothesis 4). This was corroborated by a decrease in SEPR from baseline to follow-up in the autistic EIAU group. We concluded that the autistic EIAU group at follow-up is characterized by higher neurophysiological arousal and lower stimulus-specific processing in response to target cueing compared to preschoolers receiving the A-FFIP intervention and nonautistic controls. We further conclude that this increased arousal (BPS) and attenuated stimulus-specific processing (SEPR) reduces RJA likelihood. It extends previous findings that suggested an altered arousal regulation in autism that contributes to autism-specific attentional phenomena [49, 50, 51].

Arousal regulation as mediator of intervention effects is supported by a causal mediation analysis (hypothesis 5). BPS as arousal measure mediated a group difference in RJA likelihood at follow-up between the autistic A-FFIP and EIAU group. This mediation analysis considers BPS at baseline as moderated mediation, so that the mediation by BPS is likely attributed to changes in BPS from baseline to follow-up. As a conclusion, the A-FFIP intervention might nudge arousal regulation in autistic preschoolers towards neurotypical trajectories. In contrast, the causal mediation analysis for SEPR as mediator did not converge for the developmental model, despite the strong negative association of SEPR and BPS. We speculate that the SEPR as a response measure compared to BPS as an absolute measure might have introduced insufficient variance within the given sample size to generate reliable estimators.

The interpretation of findings is limited by the small correlation of the RJA likelihood measure in eye-tracking with the RJA behavioral observation measure in autistic preschoolers [62]. We speculate that a more narrow skill set is required for eye-tracking RJA paradigms (e.g.: gaze control) compared to live-interaction RJA (e.g.: body motor control), which contribute to different performances between tasks. However, we showed that the eye-tracking measure provides a time-economic and highly-standardized assessment of RJA that is of prognostic and diagnostic relevance, while a translation to live social interactions requires further investigations [69]. In addition, we would have liked to investigate the effect of RJA changes on language development. However, our assessment of language in preschoolers with a comparatively low cognitive ability (see Table 1) by either Bayley-III or WPPSI-III provided a skewed distribution. As future research, we would encourage the development of a language test in preschoolers that can be applied across the cognitive ability range. Finally, the current analyses were not preregistered and thus the conclusions remain exploratory and require confirmation in an independent sample.

The current study emphasizes a positive effect of an NDBI program (A-FFIP) on reactive joint attention (RJA) that cascades on improved social responsiveness in preschoolers two years after the end of the A-FFIP early intervention. Additional cascades on socio-cognitive development might be observed in a higher-powered sample (see Fig. 3). Arousal regulation is concluded as promising mediating mechanism in RJA likelihood that might be steered towards neurotypical trajectories by the A-FFIP early intervention. It emphasizes pupillometry as potential neurophysiological process-outcome marker in future intervention studies.

## Electronic supplementary material

Below is the link to the electronic supplementary material.

Supplementary Material 1

## Data Availability

Eye tracking is available on reasonable request. Data preprocessing and analysis scripts are provided here: https://github.com/nicobast/BOSCA_dataanalysis.

## References

[1] Scaife M, Bruner JS (1975) The capacity for joint visual attention in the infant. Nature 253(5489):265–266. 10.1038/253265a0 1113842 10.1038/253265a0

[2] Falck-Ytter T, Kleberg JL, Portugal AM, Thorup E (2022) Social attention: developmental foundations and relevance for autism spectrum disorder. Biol Psychiatry. 10.1016/j.biopsych.2022.09.035 36639295 10.1016/j.biopsych.2022.09.035

[3] Mundy P, Newell L (2007) Attention, joint attention, and social cognition. Curr Dir Psychol Sci 16(5):269–274. 10.1111/j.1467-8721.2007.00518.x 19343102 10.1111/j.1467-8721.2007.00518.x PMC2663908

[4] Klin A, Jones W, Schultz R, Volkmar F (2003) The enactive Mind, or from actions to cognition: lessons from autism. Philos Trans R Soc Lond B Biol Sci 358(1430):345–360. 10.1098/rstb.2002.1202 12639332 10.1098/rstb.2002.1202 PMC1693114

[5] Mundy P, Block J, Delgado C, Pomares Y, Van Hecke AV, Parlade MV (2007) Individual differences and the development of joint attention in infancy. Child Dev 78(3):938–954. 10.1111/j.1467-8624.2007.01042.x 17517014 10.1111/j.1467-8624.2007.01042.x PMC2654237

[6] Gredebäck G, Fikke L, Melinder A (2010) The development of joint visual attention: a longitudinal study of gaze following during interactions with mothers and strangers. Dev Sci 13(6):839–848. 10.1111/j.1467-7687.2009.00945.x 20977555 10.1111/j.1467-7687.2009.00945.x

[7] Charman T (2003) *Why is joint attention a pivotal skill in autism?* Philosophical transactions of the Royal society of London. Ser B: Biol Sci 358(1430):315–324. 10.1098/rstb.2002.1199 10.1098/rstb.2002.1199 PMC1693124 12639329

[8] Brooks R, Meltzoff AN (2015) Connecting the Dots from infancy to childhood: A longitudinal study connecting gaze following, Language, and explicit theory of Mind. J Exp Child Psychol 130:67–78. 10.1016/j.jecp.2014.09.010 25462032 10.1016/j.jecp.2014.09.010 PMC7089676

[9] Masten AS, Cicchetti D (2010) Developmental cascades. Dev Psychopathol 22(3):491–495. 10.1017/S0954579410000222 20576173 10.1017/S0954579410000222

[10] Leekam SR, López B, Moore C (2000) Attention and joint attention in preschool children with autism. Dev Psychol 36(2):261. 10.1037/0012-1649.36.2.261 10749083 10.1037//0012-1649.36.2.261

[11] Mundy P, Sigman M, Kasari C (1994) Joint attention, developmental level, and symptom presentation in autism. Dev Psychopathol 6(3):389–401. 10.1017/S0954579400006003

[12] Guillon Q, Hadjikhani N, Baduel S, Roge B (2014) Visual social attention in autism spectrum disorder: insights from eye tracking studies. Neurosci Biobehav Rev 42:279–297. 10.1016/j.neubiorev.2014.03.013 24694721 10.1016/j.neubiorev.2014.03.013

[13] Frost KM, Pomales-Ramos A, Ingersoll B (2024) Brief report: response to joint attention and object imitation as predictors of expressive and receptive Language growth rate in young children on the autism spectrum. J Autism Dev Disord 54(3):1213–1220. 10.1007/s10803-022-05567-2 35657445 10.1007/s10803-022-05567-2 PMC10762693

[14] Bottema-Beutel K (2016) Associations between joint attention and Language in autism spectrum disorder and typical development: A systematic review and meta-regression analysis. Autism Res 9(10):1021–1035. 10.1002/aur.1624 27059941 10.1002/aur.1624

[15] Bottema-Beutel K, Kim SY, Crowley S (2019) A systematic review and meta-regression analysis of social functioning correlates in autism and typical development. Autism Res 12(2):152–175. 10.1002/aur.2055 30575308 10.1002/aur.2055

[16] Brandone AC, Stout W (2023) The origins of theory of Mind in infant social cognition: investigating longitudinal pathways from intention Understanding and joint attention to preschool theory of Mind. J Cognition Dev 24(3):375–396. 10.1080/15248372.2022.2146117 10.1080/15248372.2022.2146117 PMC10348704 37456364

[17] Lasch C, Carlson SM, Elison JT (2023) Responding to joint attention as a developmental catalyst: longitudinal associations with Language and social responsiveness. Infancy 28(2):339–366. 10.1111/infa.12515 36404295 10.1111/infa.12515 PMC9899317

[18] Charman T, Baron-Cohen S, Swettenham J, Baird G, Cox A, Drew A (2000) Testing joint attention, imitation, and play as infancy precursors to Language and theory of Mind. Cogn Dev 15(4):481–498. 10.1016/S0885-2014(01)00037-5

[19] Sandbank M, Bottema-Beutel K, Crowley S, Cassidy M, Dunham K, Feldman JI, Crank J, Albarran SA, Raj S, Mahbub P, Woynaroski TG (2020) Project AIM: autism intervention meta-analysis for studies of young children. Psychol Bull 146(1):1–29. 10.1037/bul0000215 31763860 10.1037/bul0000215 PMC8783568

[20] Dawson G, Toth K, Abbott R, Osterling J, Munson J, Estes A, Liaw J (2004) Early social attention impairments in autism: social orienting, joint attention, and attention to distress. Dev Psychol 40(2):271 14979766 10.1037/0012-1649.40.2.271

[21] Murza KA, Schwartz JB, Hahs-Vaughn DL, Nye C (2016) Joint attention interventions for children with autism spectrum disorder: a systematic review and meta-analysis. Int J Lang Communication Disorders 51(3):236–251. 10.1111/1460-6984.12212 10.1111/1460-6984.12212 26952136

[22] Schreibman L, Dawson G, Stahmer AC, Landa R, Rogers SJ, McGee GG, Kasari C, Ingersoll B, Kaiser AP, Bruinsma Y, McNerney E, Wetherby A, Halladay A (2015) Naturalistic developmental behavioral interventions: empirically validated treatments for autism spectrum disorder. J Autism Dev Disord 45(8):2411–2428. 10.1007/s10803-015-2407-8 25737021 10.1007/s10803-015-2407-8 PMC4513196

[23] Sandbank M, Bottema-Beutel K, LaPoint SC, Feldman JI, Barrett DJ, Caldwell N, Dunham K, Crank J, Albarran S, Woynaroski T (2023) Autism intervention meta-analysis of early childhood studies (Project AIM): updated systematic review and secondary analysis. BMJ 383:e076733. 10.1136/bmj-2023-076733 37963634 10.1136/bmj-2023-076733 PMC10644209

[24] Shih W, Shire S, Chang Y-C, Kasari C (2021) Joint engagement is a potential mechanism leading to increased initiations of joint attention and downstream effects on Language: JASPER early intervention for children with ASD. J Child Psychol Psychiatry 62(10):1228–1235. 10.1111/jcpp.13405 33768537 10.1111/jcpp.13405 PMC9879144

[25] Kitzerow J, Hackbusch M, Jensen K, Kieser M, Noterdaeme M, Fröhlich U, Taurines R, Geissler J, Wolff N, Roessner V, Bast N, Teufel K, Kim Z, Freitag C.M. (2020) Study protocol of the multi-centre, randomised controlled trial of the Frankfurt early intervention programme A-FFIP versus early intervention as usual for toddlers and preschool children with autism spectrum disorder (A-FFIP study). Trials 21(1):1–17. 10.1186/s13063-019-3881-7 10.1186/s13063-019-3881-7 PMC7038602 32093772

[26] Mundy P, Delgado C, Block J, Venezia M, Hogan A, Seibert J (2003) Early social communication scales (ESCS). University of Miami, Coral Gables, FL

[27] Navab A, Gillespie-Lynch K, Johnson SP, Sigman M, Hutman T (2012) Eye-Tracking as a measure of responsiveness to joint attention in infants at risk for autism. Infancy 17(4):416–431. 10.1111/j.1532-7078.2011.00082.x 23284270 10.1111/j.1532-7078.2011.00082.x PMC3532884

[28] Stallworthy IC, Lasch C, Berry D, Wolff JJ, Pruett JR Jr., Marrus N, Swanson MR, Botteron KN, Dager SR, Estes AM, Hazlett HC, Schultz RT, Zwaigenbaum L, Piven J, Elison JT (2022) Variability in responding to joint attention cues in the first year is associated with autism outcome. J Am Acad Child Adolesc Psychiatry 61(3):413–422. 10.1016/j.jaac.2021.03.023 33965519 10.1016/j.jaac.2021.03.023 PMC8636536

[29] Parsons JP, Bedford R, Jones EJH, Charman T, Johnson MH, Gliga T (2019) Gaze following and attention to objects in infants at Familial risk for ASD. Front Psychol 10. https://www.frontiersin.org/journals/psychology/articles/10.3389/fpsyg.2019.01799 10.3389/fpsyg.2019.01799 PMC6710391 31481909

[30] Bedford R, Elsabbagh M, Gliga T, Pickles A, Senju A, Charman T, Johnson MH (2012) Precursors to social and communication difficulties in infants At-Risk for autism: gaze following and attentional engagement. J Autism Dev Disord 42(10):2208–2218. 10.1007/s10803-012-1450-y 22278030 10.1007/s10803-012-1450-y

[31] Falck-Ytter T, Thorup E, Bölte S (2015) Brief report: lack of processing Bias for the objects other people attend to in 3-Year-Olds with autism. J Autism Dev Disord 45(6):1897–1904. 10.1007/s10803-014-2278-4 25331324 10.1007/s10803-014-2278-4 PMC4441907

[32] Jones W, Klin A (2009) Heterogeneity and homogeneity across the autism spectrum: the role of development. J Am Acad Child Adolesc Psychiatry 48(5):471–473 19395902 10.1097/CHI.0b013e31819f6c0d

[33] Bell A, Jones K (2015) Explaining fixed effects: random effects modeling of Time-Series Cross-Sectional and panel data. Political Sci Res Methods 3(1):133–153. 10.1017/psrm.2014.7

[34] Moscatelli A, Mezzetti M, Lacquaniti F (2012) Modeling psychophysical data at the population-level: the generalized linear mixed model. J Vis 12(11):26–26. 10.1167/12.11.26 23104819 10.1167/12.11.26

[35] Johnson MH, Charman T, Pickles A, Jones EJH (2021) Annual research review: anterior modifiers in the emergence of neurodevelopmental disorders (AMEND)-a systems neuroscience approach to common developmental disorders. J Child Psychol Psychiatry 62(5):610–630. 10.1111/jcpp.13372 33432656 10.1111/jcpp.13372 PMC8609429

[36] Laeng B, Sirois S, Gredebäck G (2012) Pupillometry A window to the preconscious?? Perspect Psychol Sci 7(1):18–27 26168419 10.1177/1745691611427305

[37] Mathôt S, Fabius J, Van Heusden E, Van der Stigchel S (2018) Safe and sensible preprocessing and baseline correction of pupil-size data. Behav Res Methods 50(1):94–106. 10.3758/s13428-017-1007-2 29330763 10.3758/s13428-017-1007-2 PMC5809553

[38] Kret ME, Sjak-Shie EE (2018) Preprocessing pupil size data: guidelines and code. Behav Res Methods. 10.3758/s13428-018-1075-y 10.3758/s13428-018-1075-y PMC6538573 29992408

[39] Nassar MR, Rumsey KM, Wilson RC, Parikh K, Heasly B, Gold JI (2012) Rational regulation of learning dynamics by pupil-linked arousal systems. Nat Neurosci 15(7):1040–1046. 10.1038/nn.3130 22660479 10.1038/nn.3130 PMC3386464

[40] Mather M, Clewett D, Sakaki M, Harley CW (2016) Norepinephrine ignites local hotspots of neuronal excitation: how arousal amplifies selectivity in perception and memory. Behav Brain Sci 39:e200–e200. 10.1017/S0140525X15000667 26126507 10.1017/S0140525X15000667 PMC5830137

[41] Aston-Jones G, Cohen JD (2005) An integrative theory of locus coeruleus-norepinephrine function: adaptive gain and optimal performance. Annu Rev Neurosci 28:403–450. 10.1146/annurev.neuro.28.061604.135709 16022602 10.1146/annurev.neuro.28.061604.135709

[42] Gilzenrat MS, Nieuwenhuis S, Jepma M, Cohen JD (2010) *Pupil diameter tracks changes in control state predicted by the adaptive gain theory of locus coeruleus function.* Cognitive. Behav Neurosci 10(2):252–269. 10.3758/cabn.10.2.252. Affective 10.3758/CABN.10.2.252 PMC3403821 20498349

[43] Zhao S, Chait M, Dick F, Dayan P, Furukawa S, Liao H-I (2019) Pupil-linked phasic arousal evoked by violation but not emergence of regularity within rapid sound sequences. Nat Commun 10(1):4030–4030. 10.1038/s41467-019-12048-1 31492881 10.1038/s41467-019-12048-1 PMC6731273

[44] Cole L, Lightman S, Clark R, Gilchrist ID (2022) Tonic and phasic effects of reward on the pupil: implications for locus coeruleus function. Proceedings of the Royal Society B: Biological Sciences 289(1982):20221545 10.1098/rspb.2022.1545 10.1098/rspb.2022.1545 PMC9470248 36100024

[45] Boxhoorn S, Bast N, Super H, Polzer L, Cholemkery H, Freitag CM (2020) Pupil dilation during visuospatial orienting differentiates between autism spectrum disorder and attention-deficit/hyperactivity disorder. J Child Psychol Psychiatry 61(5):614–624. 10.1111/jcpp.13179 31853987 10.1111/jcpp.13179

[46] Anderson CJ, Colombo J (2009) Larger tonic pupil size in young children with autism spectrum disorder. Dev Psychobiol 51(2):207–211 18988196 10.1002/dev.20352 PMC3744086

[47] Zhao S, Liu Y, Wei K (2022) Pupil-Linked arousal response reveals aberrant attention regulation among children with autism spectrum disorder. J Neurosci. 10.1523/JNEUROSCI.0223-22.2022 35641188 10.1523/JNEUROSCI.0223-22.2022 PMC9270919

[48] Bast N, Boxhoorn S, Supér H, Helfer B, Polzer L, Klein C, Cholemkery H, Freitag CM (2023) Atypical arousal regulation in children with autism but not with Attention-Deficit/Hyperactivity disorder as indicated by pupillometric measures of locus coeruleus activity. Biol Psychiatry: Cogn Neurosci Neuroimaging 8(1):11–20. 10.1016/j.bpsc.2021.04.010 33930603 10.1016/j.bpsc.2021.04.010

[49] Orekhova E, Stroganova T (2014) Arousal and attention re-orienting in autism spectrum disorders: evidence from auditory event-related potentials. Front Hum Neurosci 8(34). 10.3389/fnhum.2014.00034 10.3389/fnhum.2014.00034 PMC3915101 24567709

[50] Bast N, Poustka L, Freitag CM (2018) The locus coeruleus–norepinephrine system as pacemaker of attention–a developmental mechanism of derailed attentional function in autism spectrum disorder. Eur J Neurosci 47(2):115–125. 10.1111/ejn.13795 29247487 10.1111/ejn.13795

[51] Bellato A, Arora I, Kochhar P, Ropar D, Hollis C, Groom MJ (2023) Relationship between autonomic arousal and attention orienting in children and adolescents with ADHD, autism and co-occurring ADHD and autism. Cortex. 10.1016/j.cortex.2023.06.002 37459680 10.1016/j.cortex.2023.06.002

[52] Ishikawa M, Senju A, Kato M, Itakura S (2022) Physiological arousal explains infant gaze following in various social contexts. Royal Soc Open Sci 9(8):220592. 10.1098/rsos.220592 10.1098/rsos.220592 PMC9382202 35991332

[53] Lord C (2015) ADOS-2: diagnostische beobachtungsskala für Autistische Störungen – 2 manual. Huber, Bern

[54] Bölte S, Rühl D, Schmötzer G, Poustka F (2008) ADI-R: diagnostisches interview für autismus – Revidiert: Deutsche Fassung des autism diagnostic interview – Revised (ADI-R). Huber, Bern

[55] Kim SH, Thurm A, Shumway S, Lord C (2013) Multisite study of new autism diagnostic interview-revised (ADI-R) algorithms for toddlers and young preschoolers. J Autism Dev Disord 43(7):1527–1538. 10.1007/s10803-012-1696-4 23114567 10.1007/s10803-012-1696-4 PMC3594108

[56] Polzer L, Schenk M, Raji N, Kleber S, Lemler C, Kitzerow-Cleven J, Kim Z, Freitag CM, Bast N (2024) Temporal progression of pupil dilation and gaze behavior to emotion expressions in preschoolers with autism spectrum disorder. Sci Rep 14(1):7843. 10.1038/s41598-024-58480-2 38570565 10.1038/s41598-024-58480-2 PMC10991397

[57] Plück J, Scholz K-K, Döpfner M (2022) CBCL/ 1 ½-5, C-TRF/ 1 ½-5: Deutsche Kleinkind- und Vorschulalter-Formen der Child behavior checklist von Thomas M. Achenbach und Leslie A. Rescorla: Elternfragebogen für Klein- und Vorschulkinder: Fragebogen für Erzieherinnen von Klein- und Vorschulkinder: Manual (1. Auflage). Göttingen: Hogrefe

[58] Polzer L, Freitag CM, Bast N (2022) Pupillometric measures of altered stimulus-evoked locus coeruleus‐norepinephrine activity explain attenuated social attention in preschoolers with autism spectrum disorder. Autism Res 15(11):2167–2180. 10.1002/aur.2818 36111843 10.1002/aur.2818

[59] Reuner G (2015) Bayley Skales of infant and toddler development – third edition. German version. Hogrefe, Göttingen

[60] Petermann F (2014) Wechsler preschool and primary scale of intelligence- III: (WPPSI-III); Deutsche Version (3., überarb. und erw. Aufl.). Frankfurt am Main: Pearson

[61] Sturm A, Kuhfeld M, Kasari C, Mccracken JT (2017) Development and validation of an item response theory-based social responsiveness scale short form. J Child Psychol Psychiatry 58(9):1053–1061. 10.1111/jcpp.12731 28464350 10.1111/jcpp.12731

[62] Franchini M, Glaser B, Gentaz E, Wood H, Eliez S, Schaer M (2017) The effect of emotional intensity on responses to joint attention in preschoolers with an autism spectrum disorder. Res Autism Spectr Disorders 35:13–24. 10.1016/j.rasd.2016.11.010

[63] Nyström M, Holmqvist K (2010) An adaptive algorithm for fixation, saccade, and glissade detection in Eyetracking data. Behav Res Methods 42(1):188–204 20160299 10.3758/BRM.42.1.188

[64] Kästel IS, Vllasaliu L, Wellnitz S, Cholemkery H, Freitag CM, Bast N (2021) Repetitive behavior in children and adolescents: psychometric properties of the German version of the repetitive behavior scale-revised. J Autism Dev Disord 51:1224–1237. 10.1007/s10803-020-04588-z 32642960 10.1007/s10803-020-04588-z

[65] Robins JM, Greenland S (1992) Identifiability and exchangeability for direct and indirect effects. Epidemiology 3(2):143–155 1576220 10.1097/00001648-199203000-00013

[66] Imai K, Keele L, Tingley D (2010) A general approach to causal mediation analysis. Psychol Methods 15(4):309–334. 10.1037/a0020761 20954780 10.1037/a0020761

[67] Nakagawa S, Schielzeth H (2013) A general and simple method for obtaining R2 from generalized linear mixed-effects models. Methods Ecol Evol 4(2):133–142

[68] Senju A, Csibra G (2008) Gaze following in human infants depends on communicative signals. Curr Biol 18(9):668–671. 10.1016/j.cub.2008.03.059 18439827 10.1016/j.cub.2008.03.059

[69] Nyström P, Thorup E, Bölte S, Falck-Ytter T (2019) Joint attention in infancy and the emergence of autism. Biol Psychiatry 86(8):631–638. 10.1016/j.biopsych.2019.05.006 31262432 10.1016/j.biopsych.2019.05.006

[70] Lee T-H, Greening SG, Ueno T, Clewett D, Ponzio A, Sakaki M, Mather M (2018) Arousal increases neural gain via the locus coeruleus–noradrenaline system in younger adults but not in older adults. Nat Hum Behav 2(5):356–366. 10.1038/s41562-018-0344-1 30320223 10.1038/s41562-018-0344-1 PMC6176734

[71] Dahl MJ, Mather M, Werkle-Bergner M (2022) Noradrenergic modulation of rhythmic neural activity shapes selective attention. Trends Cogn Sci 26(1):38–52. 10.1016/j.tics.2021.10.009 34799252 10.1016/j.tics.2021.10.009 PMC8678372

[72] Pfeffer T, Keitel C, Kluger DS, Keitel A, Russmann A, Thut G, Donner TH, Gross J (2022) Coupling of pupil- and neuronal population dynamics reveals diverse influences of arousal on cortical processing. eLife 11:e71890. 10.7554/eLife.71890 35133276 10.7554/eLife.71890 PMC8853659

[73] Kim Y, Kadlaskar G, Keehn RM, Keehn B (2022) Measures of tonic and phasic activity of the locus coeruleus—norepinephrine system in children with autism spectrum disorder: an event-related potential and pupillometry study. Autism Res 15(12):2250–2264. 10.1002/aur.2820 36164264 10.1002/aur.2820 PMC9722557

