# Supplementary material for: Early intervention increases reactive joint attention in autistic preschoolers with arousal regulation as mediator
Source: Eur Child Adolesc Psychiatry. 2025 May 10;34(10):3161–74. doi: 10.1007/s00787-025-02738-1 (PMC12592278; doi:10.1007/s00787-025-02738-1)
Supplement: Supplementary file 1 — Supplementary Material 1 [file 787_2025_2738_MOESM1_ESM.docx]

Figure S1. Date of assessments between groups.


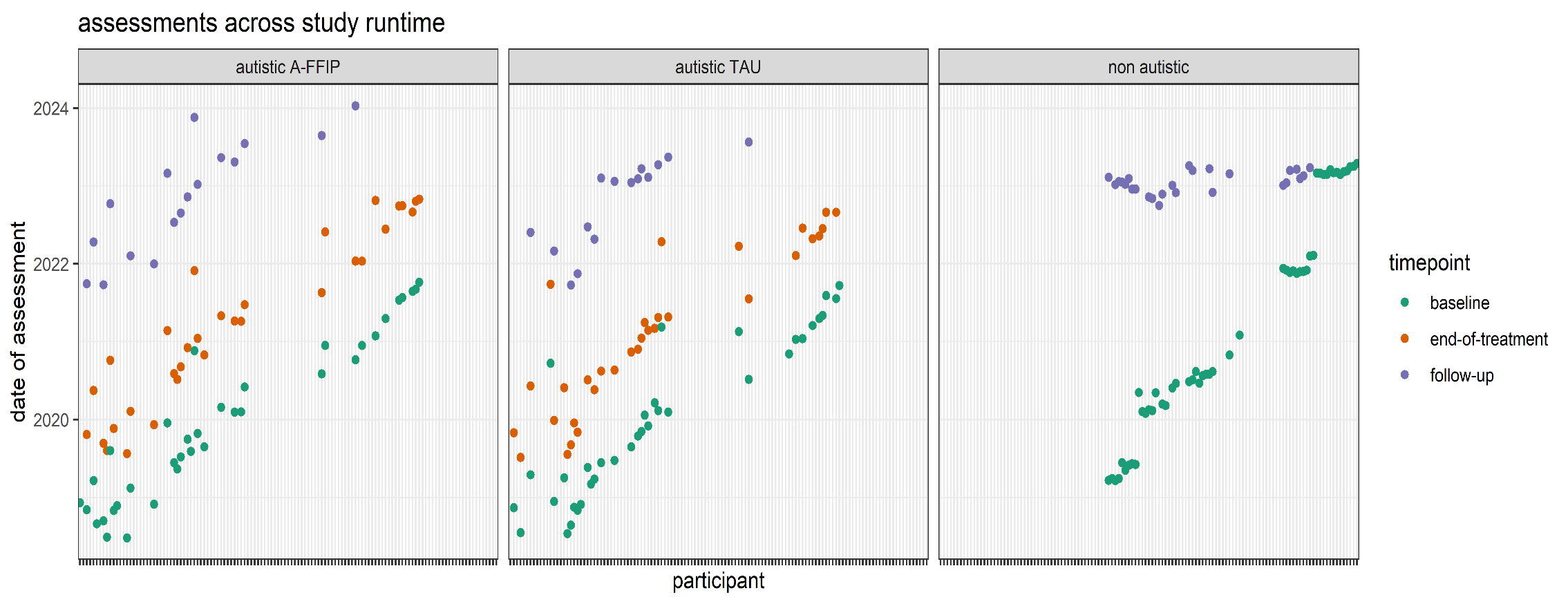


Figure S2. Distribution of the participant random intercept in the intervention (top) and developmental model (bottom).


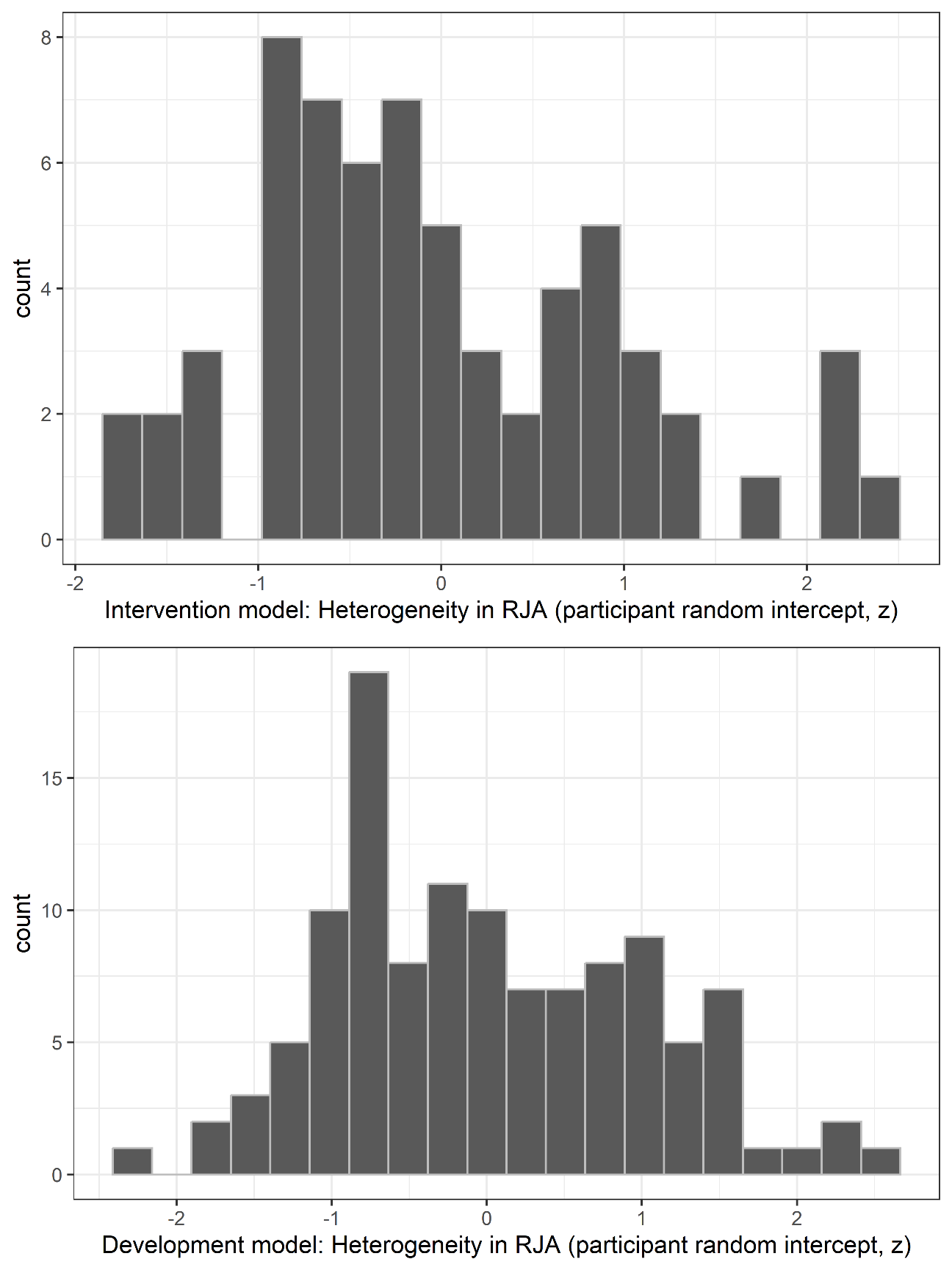


Table S1.Models of the dropout analysis.

| A.) RJA likelihood: Effect of retention status and autistic group at baseline. | | | | | |
| --- | --- | --- | --- | --- | --- |
|  | DF | Sum Sq | Mean Sq | F-value | p-value |
| retention status (RS) | 1 | 0.05 | 0.05 | 0.01 | 0.94 |
| autistic group (G) | 1 | 27.67 | 27.67 | 2.71 | 0.11 |
| RS x G | 1 | 6.07 | 6.07 | 0.59 | 0.44 |
| Residuals | 56 | 572.53 | 10.22 | NA | NA |

| B.) Autism symptom severity: Effect of retention status and autistic group at baseline. | | | | | |
| --- | --- | --- | --- | --- | --- |
|  | DF | Sum Sq | Mean Sq | F-value | p-value |
| retention status (RS) | 1 | 0.41 | 0.41 | 0.17 | 0.68 |
| autistic group (G) | 1 | 2.22 | 2.22 | 0.90 | 0.35 |
| RS x G | 1 | 0.46 | 0.46 | 0.19 | 0.67 |
| Residuals | 56 | 137.89 | 2.46 | NA | NA |

| C.) Comorbid Psychopathology: Effect of retention status and autistic group at baseline. | | | | | |
| --- | --- | --- | --- | --- | --- |
|  | DF | Sum Sq | Mean Sq | F-value | p-value |
| retention status (RS) | 1 | 0.01 | 0.01 | 0.00 | 0.99 |
| autistic group (G) | 1 | 159.78 | 159.78 | 1.89 | 0.18 |
| RS x G | 1 | 0.24 | 0.24 | 0.00 | 0.96 |
| Residuals | 49 | 4134.95 | 84.39 | NA | NA |

| D.) Data quality as missing data: Effect of retention status and autistic group at baseline. | | | | | |
| --- | --- | --- | --- | --- | --- |
|  | DF | Sum Sq | Mean Sq | F-value | p-value |
| retention status (RS) | 1 | 0.00 | 0.00 | 0.04 | 0.85 |
| autistic group (G) | 1 | 0.02 | 0.02 | 1.50 | 0.23 |
| RS x G | 1 | 0.01 | 0.01 | 0.45 | 0.50 |
| Residuals | 51 | 0.59 | 0.01 | NA | NA |
